# Ontario

**Published:** 1897-08

**Authors:** 


*Ontario.* Bacteriologist Mackenzie, of the Provincial Board of Health of Ontario, referring to the outbreaks of rabies, says of its origin, that it is more likely due to the introduction of imported dogs, chiefly from the United States. The destruction of masterless dogs, and the enforcement of a muzzling-law for some months after an outbreak were recommended as sufficient for its control.

